# Regional Therapies Utilized in Treating Unresectable Colorectal Adenocarcinoma with Peritoneal Metastases

**DOI:** 10.3390/cancers18050863

**Published:** 2026-03-07

**Authors:** Shray Malik, Vanessa Le, Farshid Dayyani, Maheswari Senthil, Oliver S. Eng, Michael P. O’Leary

**Affiliations:** 1 Department of Surgery, University of California-Irvine, Orange, CA 92868, USA; vanesl11@hs.uci.edu (V.L.); maheswas@hs.uci.edu (M.S.); oeng@hs.uci.edu (O.S.E.); olearym@hs.uci.edu (M.P.O.); 2 Department of Medicine, Chao Family Comprehensive Cancer Center, University of California-Irvine, Orange, CA 92868, USA; fdayyani@hs.uci.edu

**Keywords:** colorectal peritoneal metastases, locoregional therapy, unresectable peritoneal carcinomatosis

## Abstract

Colorectal peritoneal metastases portend a poor prognosis when compared to other isolated sites of metastatic disease. The advent of regional therapies, including cytoreductive surgery, have improved outcomes for patients with peritoneal carcinomatosis. However, these options are typically only available in patients deemed to have resectable disease. For patients with unresectable peritoneal disease, non-surgical regional therapy has only been studied in early-phase clinical trials. This represents a gap in therapy in a population with a desperate need. In this review, we highlight the current limited data, as well as postulate on the future direction of regional therapies in patients with unresectable peritoneal metastases from colorectal adenocarcinoma.

## Simple Summary

Metastatic spread of colorectal cancer to the peritoneal lining is a difficult disease to treat and often has poor outcomes. Some patients may benefit from surgery and heated chemotherapy delivered directly into the abdomen; however, many patients have disease that is unresectable. For these patients, treatment options are limited, and standard systemic chemotherapy has reduced effectiveness. This review examines the multitude of regional treatments that deliver therapy directly into the abdominal cavity for patients with unresectable disease. These approaches aim to control symptoms, slow disease progression, and in some cases reduce tumor burden enough to allow future cytoreductive surgery. We summarize early clinical studies evaluating these therapies and discuss their potential benefits and limitations.

## 1. Introduction

Colorectal cancer (CRC) is the third-most diagnosed cancer in the world and is second in mortality, with a global incidence of 1.9 million cases annually and a worldwide death rate of 935,000 [1]. Despite population-level changes towards healthier lifestyles and early screening, it remains one of the most common malignancies in the world. Approximately 20% of patients with newly diagnosed CRC will present with stage IV disease at the time of diagnosis. Peritoneal carcinomatosis (PC) represents malignant metastatic spread along the surface of the peritoneal lining. In population-based studies, 5–10% of patients with CRC had synchronous PC while metachronous PC can develop in up to half of patients in their disease course [2]. PC was the first and only site of metastases in 4.8% of patients [3]. While PC from CRC is not common as the isolated site of metastatic spread, it portends a dismal outcome with 5-year overall survival of 8% [4].

There have been tremendous therapeutic advancements over the past several decades in CRC treatment due to improvements in chemotherapy regimens and the development of targeted therapies [5]. However, in patients with metastatic CRC, peritoneal disease remains an aggressive disease with a reduced overall survival compared with patients with nonperitoneal metastases [6]. Regional therapy options including cytoreduction and heated intraperitoneal chemotherapy (HIPEC) have largely remained unchanged and the survival in isolated peritoneal metastatic disease to date has shown overall survival improvement with cytoreduction alone. In the setting of oligometastatic disease, peritoneal disease has the worst overall survival at 16.3 months, compared to 19.1 months in patients with liver metastasis, and 24.6 months in patients with lung metastasis [6]. Additionally, peritoneal carcinomatosis presents several unique challenges including severe symptoms with limited available palliative treatments for high volume disease, difficulty in detecting low volume PC with conventional imaging modalities, and a limited response to systemic chemotherapy [7,8,9].

The introduction and advancement of regional therapies have shown modest improvement in outcomes for patients with colorectal PC. When considering resectable disease, complete cytoreduction has been the best prognosticator for improved overall survival. However, patient selection is critical when considering who would benefit from CRS with or without HIPEC. One such criterion is the extent of metastatic disease and feasibility of complete cytoreduction. The number of patients potentially eligible for a regional therapeutic approach is limited, as 23% of all laparotomies for peritoneal carcinomatosis are eventually declared as unresectable [10]. For patients with unresectable disease, systemic chemotherapy is often the first-line, and only-line treatment; however, the peritoneal–plasma barrier greatly limits its therapeutic effects [11]. The treatment options for unresectable PC remain limited and represent a gap in management in a population who desperately need additional options.

At present, there is limited data on the use of regional therapies in patients with colorectal cancer with unresectable or incompletely resected peritoneal disease. When considering the use of regional therapies for these patients, the primary goal can be categorized into one of three categories: palliative symptom control, prolonging survival, and converting unresectable disease into resectable disease. Although the data is extremely limited, some small retrospective studies have shown that certain regional therapies may reduce the volume of malignant ascites and prolong control of carcinomatosis-related symptoms [12,13]. Additional studies have explored potential survival benefit and, in the most ideal scenario, the possibility of conversion to resectable disease. The purpose of this article is to review the existing literature on this topic and discuss the potential role of regional therapies in improving outcomes in patients with unresectable PC.

## 2. Regional Therapies

### 2.1. Cytoreductive Surgery and Hyperthermic Intraperitoneal Chemotherapy (HIPEC)

The concept of cytoreductive surgery (CRS) and hyperthermic intraperitoneal chemotherapy (HIPEC) to treat peritoneal carcinomatosis was pioneered in the 1990s [14,15]. Cytoreductive surgery is a procedure that entails reducing the tumor burden within the abdominal cavity by removing all visible signs of metastatic disease. When combined with HIPEC, which involves infusing chemotherapy warmed to 42 °C into the intraperitoneal cavity for a period of time, a profound survival benefit was historically noted in patients with colorectal PC when compared to standard treatment [16]. The index prospective randomized clinical trial by Verwaal et al. included 105 patients who were randomly assigned to receive either systemic chemotherapy with or without palliative surgery or aggressive cytoreductive surgery with HIPEC for resectable CRPM. After a median follow-up period of 21.6 months, the median survival was 12.6 months in the control arm and 22.3 months in the CRS/HIPEC arm [17]. A long-term follow-up study of the same cohort of patients revealed a 5-year survival rate of 45% for patients who successfully underwent a complete cytoreduction [18]. Multiple other studies show a survival benefit for patients who are able to undergo complete CRS [19,20,21,22,23]. While these studies have shown encouraging results, many patients are deemed to have inoperable/unresectable disease which has historically precluded them from undergoing cytoreductive surgery with HIPEC.

At present, there are no clinical trials examining the utility of palliative CRS and HIPEC in unresectable PC from a colorectal primary. Aggressive surgical debulking introduces increased morbidity and mortality and will ultimately delay or prohibit initiation of standard of care systemic chemotherapy. Additional perspectives on the utility of an aggressive regional approach can be drawn from other primary sites of PC. While more favorable outcomes have been observed with appendiceal adenocarcinoma with CRS and HIPEC, unresectable disease in both appendiceal and colorectal PC have commonalities. One prospective trial by Berger et al. examined the feasibility of using iterative HIPEC (IHIPEC) for unresectable disease in patients with high-grade appendiceal carcinoma. Over the 3-year span of the study, 31 patients with high-grade appendiceal ex-goblet cell adenocarcinoma with high-burden unresectable peritoneal metastases were allocated to IHIPEC (*n* = 7), systemic chemotherapy alone (*n* = 17), or curative intent CRS/HIPEC (*n* = 7). Patients in the first group underwent IHIPEC with mitomycin C at 6-week intervals for a total of three cycles following systemic chemotherapy. Median overall survival after IHIPEC was better compared with a matched group of patients receiving systemic chemotherapy (24.6 vs. 7.9 months; *p*  =  0.005), and similar to those who underwent CRS/HIPEC (24.6 vs. 16.5 months; *p*  =  0.62) [24]. This early-phase study showed some promising results in OS when comparing IHIPEC to standard of care systemic chemotherapy alone. However, it should be noted that goblet cell carcinoma (GCC) exhibits distinct biological differences when compared to colorectal adenocarcinoma, including differences in metastatic patterns and chemotherapy responsiveness. This data is included to illustrate potential regional treatment strategies rather than to imply biological equivalence. Outcomes in appendiceal or GCC cohorts should therefore be interpreted cautiously when extrapolated to colorectal adenocarcinoma.

In cases of unresectable disease, patients can present with malignant bowel obstruction, symptomatic ascites, and/or large abdominal masses. Surgeons are often challenged in determining how to manage these patients: whether to palliate symptoms with an intervention or to initiate systemic therapy. A number of variables influence this challenging decision. Surgical interventions including palliative bypass may not be technically possible and there is conflicting data surrounding incomplete cytoreduction in patients with peritoneal metastases. The longstanding dogma that an incomplete cytoreduction does not confer a survival benefit is based on retrospective data. Sugarbaker et al. showed that incomplete or palliative CRS in patients with colon or rectal cancer with biopsy-confirmed peritoneal metastases at the time of CRS have poor outcomes [25]. In a retrospective study of CRS and HIPEC, eighty-five patients (34.1%) were identified to have undergone incomplete cytoreduction for metastatic CRC with peritoneal involvement. HIPEC/EPIC was used in all but 21 patients who underwent CC-3 resection (palliative surgery). Thirty-three patients (38.8%) underwent a CC-2 resection (residual tumor nodules 0.25–2.5 cm). A greater surgical effort was seen in the CC-1 resection group (residual tumor nodules < 0.25 cm), with the percentage of patients having a 9 h surgery or greater being 65.5% for the CC-1, 37.9% for the CC-2, and 17.6% for the CC-3 group [25]. CC-1 resection had a 38.7% 2-year overall survival compared to a 15.1% and 4.8% 2-year overall survival for a CC-2 or CC-3 resection, respectively. From this data, there is no survival benefit from aggressive surgical debulking while leaving gross residual disease.

A systematic review article published by Heaney et al. aimed to examine the outcomes of patients who underwent incomplete cytoreduction with intraperitoneal chemotherapy for colorectal peritoneal metastases (CRPMs). This review included data from 19 papers including 15 case series, 3 case–control studies, and 1 randomized control trial [26]. In the 19 studies analyzed, a total of 2790 patients underwent CRS and intraperitoneal chemotherapy for CRPM. 986 (35%) patients underwent incomplete cytoreduction with final resection scores of CC-2 or CC-3. Median survival in the complete cytoreduction group ranged from 11 to 62 months while survival in the incomplete cytoreduction group ranged from 2.4 to 32 months. A total of 331 patients who underwent incomplete cytoreduction received intraperitoneal chemotherapy and survival in this cohort ranged from 4.5 to 32 months [26]. This study suggests that incomplete cytoreduction, with or without intraperitoneal chemotherapy, does not confer a survival benefit.

It is well established in the literature that the completeness of cytoreduction has a strong prognostic impact on the survival of patients with PC of colorectal origin. However, the data regarding the utility of palliative HIPEC, iterative HIPEC, and incomplete CRS on patients with unresectable CRPM continues to show poor OS. Data from selected studies are summarized in Table 1. There is a tremendous void in regional therapeutic options in this challenging patient population. Existing data suggests that less invasive regional interventions should be aimed at palliating symptoms while limiting potential morbidity.

### 2.2. Normothermic Intraperitoneal Chemotherapy (NIPEC)

With the foundational understanding that an aggressive surgical approach in unresectable CPRM is unlikely to be beneficial, less invasive interventions aimed to palliate symptoms or bridge to resectability need to be explored in the context of clinical trials. One such regional therapy called normothermic intraperitoneal chemotherapy (NIPEC) involves the instillation of intraperitoneal chemotherapy at body temperature. As compared to HIPEC, which is administered intraoperatively, NIPEC can be utilized long-term (NIPEC-LT) via an intraperitoneal port [27]. It has been used in both the neoadjuvant and adjuvant setting and can be given as a bidirectional therapy with systemic IV chemotherapy [28]. Its efficacy has been studied primarily in ovarian cancer, gastric cancer, and malignant primary peritoneal mesothelioma. In ovarian and malignant peritoneal mesothelioma, NIPEC-LT has been shown to be as effective as an adjuvant therapy [29,30].

Given the improved outcomes in other malignancies, new and ongoing research has been examining NIPEC as a regional therapy in CRPM. An interventional study by Murono et al. examined the safety of intraperitoneal paclitaxel (PTX) in conjunction with systemic chemotherapy for unresectable CRPM. In this phase 1 trial, patients with CRC and PC received a peritoneal access port and selected between two conventional IV chemotherapeutic regimens: 5-FU, folinic acid, oxaliplatin, and bevacizumab (mFOLFOX6-bevacizumab) (*n* = 3); or capecitabine, oxaliplatin, and bevacizumab (CapeOX-bevacizumab) (*n* = 3). In addition to the conventional chemotherapy, all six participants received weekly instillations of intraperitoneal PTX 20 mg/m^2^ for one hour and were followed for one year to observe dose-limiting toxicities (DLTs). Grade 3 adverse events were found in 66.7% of participants, and 100% of patients experienced an adverse event of any grade. There was no dose-limiting toxicity, grade 4 adverse events, or adverse events associated with the peritoneal access port [31]. A one-year follow-up study examined the response rate (RR), PFS, 1-year survival, frequency of improvement in the peritoneal cancer index (PCI), and cytology of peritoneal lavage. Of the original six-patient cohort, three were still alive and on systemic chemotherapy as of the final follow-up (approximately 3 years after the conclusion of patient enrollment). The study found a response rate of 25%, PFS of 8.8 months (with a range of 6.8–12 months), a median survival time of 29.3 months, and a 100% 1-year survival rate. Additionally, PCI score improved in 50% of the cohort, and the five patients whose peritoneal lavage were positive before chemotherapy were cytologically negative thereafter [32]. Although this study was limited by its small cohort size and single-arm nature, intraperitoneal PTX shows potential promise as a treatment for unresectable colorectal PC. A phase II trial is underway to further examine efficacy of intraperitoneal PTX (the iPac-02 trial) [33].

The INTERACT trial is a similar study examining the efficacy of intraperitoneal irinotecan [34]. This phase 1 trial was a 3 + 3 dose escalation trial that enrolled adult patients with peritoneal metastasis from CRC, for whom CRS-HIPEC was not considered a good option. While the protocol planned for intraperitoneal irinotecan tiers of 50, 100, 200, 300, and 400 mg, during the study, it was observed that the 100 mg dose level had DLTs while the first level did not. Thus, a 75 mg dose level was added via amendment to reach the highest tolerable dose of intraperitoneal irinotecan. The study drug was administered concurrently with systemic FOLFOX and bevacizumab every two weeks for a maximum of 12 cycles. Overall, eighteen patients with a median PCI score of 28 were treated. The 75 mg dose enrolled nine patients, and no DLTs were observed. While most toxicities were grades 1 or 2, there were grade 3 (most commonly neutropenia) and grade 4 toxicities observed. Overall, the toxicity profile was similar to that of FOLFOX, though there was an elevated incidence of severe gastrointestinal adverse events compared to what is expected from FOLFOX–bevacizumab alone. A radiological response was found in most patients, including a subgroup with no viable tumor cells detected after treatment, and four patients were ultimately bridged to CRS-HIPEC. The median overall survival in this traditionally challenging patient population was 23.9 months. This suggests that intraperitoneal irinotecan may have a role as a regional therapy and may ultimately bridge unresectable patients to CRS and HIPEC [35]. A phase II trial (INTERACT-II) is currently underway with an accrual of 85 patients on a single arm of intraperitoneal irinotecan 75 mg and intravenous mFOLFOX4 with bevacizumab [36].

Based on the limited data from recent and ongoing trials evaluating NIPEC in unresectable CRPM, this treatment modality shows promise in improving overall survival and potential as a bridging therapeutic to more aggressive, curative intent CRS and HIPEC. Selected studies are summarized in Table 2. Additional larger scale studies are underway to better evaluate its safety and efficacy.

### 2.3. Pressurized Intraperitoneal Aerosol Chemotherapy (PIPAC)

Pressurized intraperitoneal aerosol chemotherapy (PIPAC) is a treatment modality first introduced in 2011 that offers an alternative method for intraperitoneal drug delivery [37]. PIPAC is a laparoscopic method for repetitive delivery of intraperitoneal chemotherapy as a pressurized aerosol, with proposed benefits including enhanced tumor penetration, homogeneous intraperitoneal drug distribution, and limited toxicity. Due to encouraging preliminary results regarding its use, PIPAC has been adopted in some centers as a treatment option for unresectable peritoneal metastatic disease.

One phase II trial including 20 patients from two Dutch tertiary care centers examined the safety and antitumor activity of PIPAC with oxaliplatin delivered every 6–8 weeks with or without concomitant systemic therapy in patients with unresectable colorectal peritoneal metastases [38]. The 20 enrolled patients underwent 59 total PIPAC procedures, with a median of 3 cycles per patient. After a median follow-up of 8 months, all patients had disease progression and 19 (95%) patients died. Median PFS and OS were 3.5 months and 8 months, respectively. Some patients showed biochemical, pathological, and cytological responses, but no radiological or clinically relevant macroscopic responses were observed [38]. Although this trial did not show a survival benefit, it is difficult to draw definitive conclusions as the trial population included patients with both resected and unresected primary malignancies in different stages of palliative treatment, along with other poor prognostic factors including high baseline PCI and signet ring histology. Major treatment-related adverse events including intraabdominal hemorrhage, pneumothorax, and liver toxicity occurred in 3 of 20 (15%) patients, including one possibly treatment-related death (sepsis of unknown origin). Minor treatment-related adverse events occurred in all patients after 57 of 59 (97%) procedures, the most common being abdominal pain (100% of patients) and nausea (65% of patients).

Another phase I trial from the United States included 12 patients. Eight patients had CRC and 4 with appendiceal cancer. They received oxaliplatin via PIPAC at 6-week intervals for a total of three treatments. Prior to the second and third treatments, 5-fluorouracil and leucovorin were administered for sensitization [39]. There were no surgical complications in any patients. The most common toxicities were gastrointestinal in nature and included abdominal pain, nausea, vomiting, distension, constipation, and diarrhea. Only two patients experienced grade 3 toxicities which were abdominal pain and anemia. The majority (58%) of patients in the study were able to undergo more than one PIPAC procedure, suggesting feasibility of undergoing multiple procedures. Median OS for patients in this trial was 12 months and median progression-free survival was 2.9 months [39]. For inclusion in this study, most patients were refractory to two lines of systemic chemotherapy; while the survival outcomes of this study offer minimal PFS benefit, this compares favorably to third-line chemotherapy options.

A larger retrospective cohort study by Hübner et al. included 256 patients with CRPM [40]. Patients were included if they received PIPAC with oxaliplatin in their treatment; however, there was significant variability in terms of prior CRS and HIPEC, PCI, and resectability. Patients were scheduled per protocol for 3 PIPAC administrations with oxaliplatin in 4-to-8-week intervals. This was given with additional cycles of systemic therapy if deemed appropriate by a multidisciplinary tumor board. The authors examined OS alongside histological, radiological, and macroscopic treatment response. PIPAC was combined with systemic chemotherapy in 133 patients (52%). In this study, median OS from initial PIPAC treatment was 9.4 and 11.5 months for the whole patient population and per protocol cohort, respectively. The findings in this study also indicated radiological response and symptoms at the third PIPAC treatment were predictive of survival [40]. However, PCI score and pathological findings were not predictive of survival.

Tidadini et al. published a separate retrospective cohort study in which 49 patients with unresectable PC (mainly of gastric and colorectal etiology) underwent a total of 100 PIPAC procedures with oxaliplatin or doxorubicin-cisplatin, administered in alternation with systemic chemotherapy [41]. The median initial PCI for the whole study population was 19. Among the 28 patients (57.1%) who received more than one PIPAC, PCI improved for 10 (37%), remained stable for 8 (29.6%), and worsened for 9 (33.4%). Median OS from the first PIPAC procedure for CRPM was 13.3 (5–17.6) months. Based on the results of multiple retrospective studies, PIPAC may delay oncological progression compared to systemic chemotherapy alone. However, these studies are limited by their retrospective nature, significant variability in inclusion criteria, and small sample sizes.

Orgad et al. examined a population of 346 patients with unresectable peritoneal surface malignancies who ultimately underwent 1200 PIPAC procedures to evaluate reasons for stopping therapy and to determine survival outcomes [42]. In their cohort, most patients had malignancies of gastric origin (43%), followed by colon (17%), ovarian (16%) and mesothelioma (9%). Patients with colon cancer undergoing PIPAC had the highest percentage of stopping treatment due to surgical complications (28%). Among these cases, the main complication was an inaccessible abdomen (55%). Patients with ultimately unresectable peritoneal surface malignancies of colorectal origin had an overall median survival of 11 months from the first PIPAC procedure. Patients that completed less than 3 PIPAC procedures also had a significantly shorter median and overall survival. The median survival from the first PIPAC in this group was 5 months vs. 9 months for the patients that completed three procedures. Median overall survival was 16 months for patients that completed more than 3 PIPAC procedures (*p* < 0.001).

Observational data has shown a reasonable safety profile for administration of PIPAC, and patients are often able to tolerate multiple cycles. However, the PFS for CRPM has been marginally better than third-line systemic therapy in chemotherapy refractory patients. More randomized trials are needed to better evaluate PIPAC as a treatment modality for these patients with limited options. Table 3 summarizes survival data and favorable prognostic factors from selected studies evaluating PIPAC.

### 2.4. Novel Therapeutics

Novel therapies are emerging for peritoneal metastases. They are uniquely tailored toward the specific constraints of the disease, such as the limited penetrance of systemic therapies and the tumor microenvironment (TME) of metastatic solid tumors.

Chimeric antigen receptor T-cell (CAR-T) therapy has been a transformative therapy in other forms of cancer, especially hematologic malignancies. However, solid tumors, such as colorectal cancer, show limited response due to low penetrance or an immunosuppressive TME [43,44]. In particular, intraperitoneal tumors have high frequencies of myeloid-derived suppressor cells (MDSCs) and regulatory T cells (Treg) [45]. To overcome some of these barriers, recent in vivo studies have investigated regional immunotherapy, namely intraperitoneal versus systemic tail vein (TV) administration of CEA-directed CAR-T cells against CEA-positive colorectal PC. In a study by Qian et al., higher doses of CAR-T cell therapy were associated with a better response (*p* < 0.01) and intraperitoneal administration was superior to TV administration in reducing tumor burden (*p* < 0.01) [46]. A study by Katz et al. yielded similar results. Furthermore, that study found that intraperitoneal CAR-T cell infusions provided protection against subcutaneous (SC) flank growth as well, an outcome that might be attributable to a phenomenon similar to the abscopal effect, as high levels of systemic IFN-γ were found without the detection of CAR-T cells in the SC tumors [47]. These results support the study of intraperitoneal CAR-T cell therapy in humans.

Another barrier to treatment of PC comes from immune evasion by the tumor. Oncolytic virotherapy (OV) has been trialed to address this barrier by using Vaccinia virus to restore peritoneal anticancer immunity. In a study utilizing JX-594 (JX), an oncolytic Vaccinia virus with GM-CSF, against intraperitoneal MC38 colon cancer cells within murine models, it was found that JX-treated mice had fewer and smaller tumor nodules compared to PBS-treated control mice, and JX treatment increased the number of intraperitoneal CD11c+ dendritic cells (DCs) by 3.0-fold, which served as a marker for the restoration of peritoneal anticancer immunity. Mice treated with the combined therapy of JX and anti-PD-1 showed better overall survival as compared to monotherapy of either treatment alone [48]. These results speak to OV’s potential to be used with other novel therapies.

As systemic therapy improves based on better understanding of tumor microenvironments, mutation directed therapies, and novel delivery mechanisms, this is an ample space for investigation for regional therapies for unresectable CRPM. Recent phase I clinical trials have shown the safety of NIPEC via intraperitoneal ports and PIPAC which can also be utilized for delivery of novel regional therapies.

## 3. Advances in Systemic Therapy

Although locoregional therapies represent an emerging and understudied treatment modality for patients with colorectal peritoneal metastases, it is important to discuss these options within the context of contemporary advances in systemic therapy, which have improved survival in selected subgroups. Molecular profiling plays an important role in therapeutic decision-making for metastatic colorectal cancer and may even be relevant in predicting response to regional therapies in patients with stage IVC colorectal peritoneal metastases [49]. RAS mutation status, BRAF mutation status, and microsatellite instability are particularly relevant molecular subtypes in the context of colorectal cancer.

RAS mutation status is an important factor in determining eligibility for anti-EGFR monoclonal antibodies as a therapeutic option. For patients with RAS wild-type disease, anti-EGFR monoclonal antibodies such as cetuximab and panitumumab have demonstrated improved response rates and survival; however, they provide no benefit in RAS-mutant tumors [50,51]. BRAF V600E mutations, present in approximately 8–10% of metastatic CRC, are associated with aggressive biology and a higher propensity for peritoneal dissemination [52]. For these patients, the BEACON study demonstrated improved overall survival with systemic encorafenib plus cetuximab compared with standard chemotherapy regimens, establishing molecularly targeted therapy as the standard of care for patients with BRAF-mutated metastatic CRC [53]. Lastly, microsatellite instability (MSI) testing has become a cornerstone of colorectal cancer management. Microsatellite instability represents a critical determinant of therapy, with MSI-high tumors demonstrating marked sensitivity to immune checkpoint inhibitors such as pembrolizumab as well as nivolumab and ipilimumab [54,55]. In contrast, microsatellite stable (MSS) tumors, which comprise the majority of CRPM, exhibit limited responsiveness to immunotherapy.

Despite advances in systemic therapy, patients with peritoneal metastatic disease often demonstrate suboptimal responses to these treatment modalities compared to those with liver or lung metastases. Tumor mutational status contributes to distinct immune microenvironments which influence responsiveness to different systemic therapies; however, the impact of molecular subtypes on response to regional therapies—particularly in unresectable disease—remains poorly defined in the literature. Integration of molecular profiling with consideration of regional therapeutic approaches may help to identify patients who could benefit from investigational intraperitoneal therapies. Genetic mutation analysis is critical in treating patients with stage IVC CRPM, as it helps determine the most appropriate therapeutic strategies. A deeper understanding of tumor biology and molecular drivers will ultimately be essential in improving outcomes for this challenging patient population.

## 4. Future of Regional Therapy

Unresectable peritoneal metastatic disease from colorectal adenocarcinoma represents a subset of patients with limited durable systemic therapeutic options. Regional therapy for unresectable peritoneal disease has been studied in pre-clinical in vivo models and early-stage clinical trials. Despite the use of IP chemotherapy for decades in a number of malignancies, the outcomes of unresectable CRPM patients remain poor. The future direction of regional therapy in unresectable solid tumor malignancy of the peritoneal cavity should focus on (1) mechanisms to improve tumor penetration, (2) immunomodulation of the tumor microenvironment, (3) conversion to resectable disease, and (4) broad application globally.

### 4.1. Mechanisms to Improve Drug Delivery and Tumor Penetration

A main driver that portends poor outcomes in patients with PC is the limited effects of systemic therapy due to the peritoneal–plasma barrier [11]. Therefore, future therapies should aim to utilize novel techniques to overcome this obstacle. Tumor priming with intravenous paclitaxel has been shown to induce the delivery and interstitial dispersion of nanomedicines [56]. This method enhances tumor cell apoptosis potential compared to normal cells, as shown in pre-clinical studies. In turn, the therapeutic effects of regional therapy are enhanced. Clinically, this has yet to be investigated in unresectable CRPM. However, the benefit of recent chemotherapy in epithelial ovarian cancer metastatic to the peritoneum who underwent CRS and HIPEC was explored in a meta-analysis by Kim et al. in 2022 [57]. They found that patients who underwent CRS within 6 months of their last systemic chemotherapy had a significant benefit in PFS and OS. This benefit of CRS and HIPEC did not apply for those who never received systemic chemotherapy or those who received it longer than 6 months [57]. While its exact mechanism is unknown, tumor priming may play a critical role in enhancing the effects of regional therapy, such as CRS and HIPEC.

### 4.2. Pressure Gradient Modifications and Aerosolized Chemotherapy

This treatment modality takes advantage of improved spatial distribution of the medications, and therefore, therapeutic delivery of chemotherapy. As previously discussed, PIPAC is a suitable delivery method for patients who are unresectable. However, to undergo therapy, the patients require general anesthesia for the purpose of laparoscopy and, therefore, the beneficial effects of pressure gradients. While ongoing and future PIPAC trials will show the therapeutic effects of this modality, there are barriers to a broad global delivery of this method. Namely, the necessity for general anesthesia, laparoscopic capability, and access to aerosolization equipment. Despite this, PIPAC appears to overcome a significant barrier to therapy: homogeneous distribution of chemotherapy within the intraperitoneal space [58].

There have been several mechanisms to modify and improve drug delivery intraperitoneally by changing the delivery composition or viscosity. One such modality is nanoparticle delivered medications. Nanoparticles, or nanomedicines, have a diameter of 1 to 1000 nm and carry the therapeutic to the target area. The largest advantage is the ability for the nanoparticles to reduce toxicity through tumor-targeting ligands [59]. One well-known agent is nanoparticle albumin-bound paclitaxel (nab-paclitaxel, Abraxane^TM^). The benefit to regional delivery of nanoparticles is that their small size prevents aggregation and loss of tumor-targeting potential with possible premature cargo release [60]. The future of regional therapeutics should take advantage of therapeutics that increase tumor kill with limited toxicity.

### 4.3. Conversion to Resectable Disease

For patients with colorectal peritoneal metastases, it is well described that a complete cytoreduction with removal of all macroscopic disease (CC-0 cytoreduction) confers a long-term survival benefit [17,22]. However, a complete macroscopic cytoreduction is typically only possible in patients deemed to have resectable disease. Regional therapies including PIPAC, NIPEC, and other novel therapeutics may provide an opportunity for downstaging of peritoneal disease into a state that permits CC-0 CRS. However, this is an area that is greatly understudied and there are currently ongoing trials that aim to examine this further.

There are several modalities by which institutions can evaluate a patient’s candidacy for cytoreductive surgery and determine whether the intraabdominal tumor burden has responded to a point where complete cytoreduction is possible. The gold standard for assessment of resectability is diagnostic laparoscopy [61]. A number of components are assessed during staging laparoscopy including the burden and length of serosal disease on the small intestine, the degree of pathologic response, changes in the character of peritoneal disease, and the Peritoneal Carcinomatosis Index (PCI). Small bowel involvement has been shown to be one of the most negative prognostic factors when determining resectability [62]. Histologic regression, assessed using the Peritoneal Regression Grading Score (PRGS), provides objective evidence of biologic response and can also be used to guide decision-making [63]. Lastly, a decrease in the PCI following treatment can suggest a conversion to resectable disease [64]. Although there is no specific PCI cutoff, it is generally accepted that a PCI score of less than 12 correlates with a significantly improved ability to achieve a CC-0 resection [21,22].

Additional adjuncts which have limited utility are the use of cross-sectional imaging and tumor marker assessments. There has been limited data examining the reliability of utilizing MRI or CT to detect pathologic response and therefore candidacy for cytoreduction. Multiple studies have demonstrated that there is a poor correlation between PCI calculations intraoperatively and radiographic estimates [65,66,67]. Although cross-sectional imaging can be a helpful adjunct for other reasons, it is generally unreliable to make assessments on a patient’s candidacy for complete cytoreduction. Similarly, although measurement of CEA level is a useful biomarker for monitoring disease biology and response to therapy, it cannot reliably predict the ability to achieve a CC-0 cytoreduction and therefore is not used as a stand-alone selection criterion for CRS/HIPEC [22,68].

Although conversion to resectability has been observed in small cohorts undergoing intraperitoneal locoregional therapy, this approach remains investigational and is not an established treatment paradigm. Tumor burden, particularly PCI and small bowel involvement, remain primary determinants of resectability, and meaningful conversion rates have not yet been established in randomized studies.

### 4.4. Broad Application Worldwide

Colorectal cancer is the third-most common new cancer diagnosis and ranks second in mortality, globally [69]. While significant research on the treatment of peritoneal disease has been performed in countries with abundant resources to conduct clinical trials on investigational therapeutics, delivery platforms, and outcomes, CRC incidence and mortality continue to grow worldwide. As one considers therapeutic modalities for regional treatment of unresectable CRC, providers must consider access to care. Access to advanced therapeutics is limited in lower socioeconomic countries [70]. Future therapies should be inexpensive, broadly accessible, effective, have limited toxicity, and ideally be deliverable in a resource limited environment.

## 5. Literature Selection and Limitations

This manuscript was designed as a focused narrative review intended to highlight regional therapies most commonly investigated within contemporary peritoneal surface oncology practice. Rather than performing a systematic review with formal inclusion and exclusion criteria, we selected therapies that represent the predominant strategies currently employed in specialized centers treating colorectal peritoneal metastases.

To accomplish this, PubMed/MEDLINE databases were searched using terms including “colorectal peritoneal metastases,” “unresectable,” “intraperitoneal chemotherapy,” “HIPEC,” “NIPEC,” “PIPAC,” and “regional therapy” through January 2026. Given the limited number of randomized trials in unresectable colorectal peritoneal metastases, priority was given to reviewing prospective studies, early-phase clinical trials, and large retrospective cohort analyses which pertained to our clinical question. We acknowledge that this narrative approach introduces selection bias, and the presence of heterogeneous patient populations among the reviewed studies limits cross-study comparisons and the interpretation of reported survival outcomes. These limitations are inherent in this review due to the scarcity of data on the use of regional therapies in unresectable colorectal peritoneal metastases. Aside from cytoreductive surgery, most regional therapies for unresectable CRPM remain investigational, supported primarily by small early-phase trials or retrospective cohorts. Therefore, conclusions regarding survival benefit must be interpreted cautiously.

## 6. Conclusions

Unresectable PC from colorectal adenocarcinoma is a challenging disease process for clinicians given the limited therapeutic options available. Regional intraperitoneal therapy is an understudied treatment modality for patients deemed to have inoperable peritoneal metastases. In this review, we highlight the existing data on the safety and efficacy of HIPEC, NIPEC, PIPAC, and other novel regional therapeutics for patients with unresectable disease. To our knowledge, there are no large-scale randomized trials to date examining any of these options for patients with unresectable colorectal PC, though there are a number on the horizon. Although early-phase clinical trial data has shown encouraging results in terms of the safety profile and even potential survival benefit, studies are limited by selection bias and low statistical power. Additional studies are needed to better assess whether there is a role for regional therapy in treating patients with unresectable colorectal PC, and future research should focus on mechanisms to improve tumor penetration, immunomodulation of the tumor microenvironment, conversion to resectable PC, and the application of this therapy globally.

## Figures and Tables

**Table 1:** Results of selected studies examining the use of CRS +/− HIPEC for patients with unresectable peritoneal carcinomatosis of colorectal or appendiceal origin.

Study	*n*	Median OS (Months)	Favorable Prognostic Factors
Berger et al. (IHIPEC) [24]	7	24.6	N/A
Berger et al. (CRS + HIPEC) [24]	7	16.5	N/A
Sugarbaker et al. (incomplete CC-1 CRS + HIPEC in colorectal adenocarcinoma) [25]	31	17	Asymptomatic
			Low or intermediate grade histology

Abbreviations: CRS = cytoreductive surgery. IHIPEC = iterative hyperthermic intraperitoneal chemotherapy. CC = completeness of cytoreduction. OS = overall survival. N/A = not available.

**Table 2:** Results of selected studies examining the use of NIPEC for patients with unresectable peritoneal carcinomatosis of colorectal or appendiceal origin.

Study	*n*	Median OS (Months)	Favorable Prognostic Factors
Murono et al. (NIPEC w/paclitaxel) [32]	6	29.3	N/A
Van Eerden et al. (NIPEC w/irinotecan) [35]	18	23.9	Subsequent CRS + HIPEC at follow-up
			75 mg irinotecan dose level

Abbreviations: NIPEC = normothermic intraperitoneal chemotherapy. 5-FU = 5-fluorouracil. OS = overall survival. CRS + HIPEC = cytoreductive surgery + hyperthermic intraperitoneal chemotherapy. N/A = not available.

**Table 3:** Results of selected studies examining the use of PIPAC for patients with unresectable peritoneal carcinomatosis of colorectal or appendiceal origin.

Study	*n*	Median OS (Months)	Favorable Prognostic Factors
Rovers et al. (PIPAC w/oxaliplatin) [38]	20	8	N/A
Raoof et al. (PIPAC w/oxaliplatin) [39]	12	12	Stable disease by RECIST criteria
Hübner et al. (PIPAC w/oxaliplatin) [40]	256	9.4	Radiologic response Symptoms at third PIPAC administration
Tidadini et al. (PIPAC w/oxaliplatin or doxorubicin-cisplatin) [41]	13	13.3	N/A
Orgad et al. (PIPAC w/cisplatin and doxorubicin or oxaliplatin) [42]	58	11	Number of PIPAC treatments
			Subsequent CRS + HIPEC at follow-up Female gender

Abbreviations: PIPAC = pressurized intraperitoneal aerosol chemotherapy. OS = overall survival. RECIST = Response Evaluation Criteria in Solid Tumors. CRS + HIPEC = cytoreductive surgery + hyperthermic intraperitoneal chemotherapy. N/A = not available.

## Data Availability

No new data were created or analyzed in this study. Data sharing is not applicable to this article.

## Abbreviations

The following abbreviations are used in this manuscript:

CRC	Colorectal cancer
PC	Peritoneal carcinomatosis
HIPEC	Heated intraperitoneal chemotherapy
CRS	Cytoreductive surgery
IHIPEC	Iterative HIPEC
GCC	Goblet cell carcinoma
EPIC	Early postoperative intraperitoneal chemotherapy
CC	Completeness of cytoreduction
CRPM	Colorectal peritoneal metastases
OS	Overall survival
NIPEC	Normothermic intraperitoneal chemotherapy
PFS	Progression-free survival
DLT	Dose-limiting toxicities
PCI	Peritoneal cancer index
PIPAC	Pressurized intraperitoneal aerosol chemotherapy
